# Do-It-Yourself Gamified Cognitive Training: Viewpoint

**DOI:** 10.2196/12130

**Published:** 2019-05-07

**Authors:** Sjors CF van de Weijer, Mark L Kuijf, Nienke M de Vries, Bastiaan R Bloem, Annelien A Duits

**Affiliations:** 1 Department of Neurology Maastricht University Medical Center Maastricht Netherlands; 2 Donders Institute for Brain, Cognition and Behaviour Department of Neurology Radboud University Medical Center Nijmegen Netherlands; 3 Department of Psychiatry and Psychology Maastricht University Medical Center Maastricht Netherlands

**Keywords:** cognitive remediation, Parkinson disease, video games

## Abstract

Cognitive decline is an important nonmotor symptom in Parkinson disease (PD). Unfortunately, very few treatment options are available. Recent research pointed to small positive effects of nonpharmacological cognitive training in PD. Most of these trainings are performed under supervision and solely computerized versions of (traditional) paper-pencil cognitive training programs, lacking rewarding gamification stimulants that could help to promote adherence. By describing 3 different self-invented ways of cognitive gaming in patients with PD, we aimed to raise awareness for the potential of gamified cognitive training in PD patients. In addition, we hoped to inspire the readers with our case descriptions, highlighting the importance of both personalization and cocreation in the development of games for health. In this viewpoint, we have presented 3 PD patients with different ages, with different disease stages, and from various backgrounds, who all used self-invented cognitive training, including elements of personalization and gamification. To indicate generalization into a larger PD population, the recruitment results from a recent cognitive game trial are added. The presented cases show similarities in terms of awareness of their cognitive decline and the ways this process could potentially be counteracted, by looking for tools to train their cognition. On the basis of the response of the recruitment procedure, there seems to be interest in gamified cognitive training in a larger PD population too. Gamification may add to traditional therapies in terms of personalization and adherence. Positive results have already been found with gamified trainings in other populations, and the cases described here suggest that PD is also an attractive area to develop and test gamified cognitive trainings. However, no results of gamified cognitive trainings in PD have been published to date. This suggests an unmet need in this area and may justify the development of gamified cognitive training and its evaluation, for which our considerations can be used.

## Introduction

### Background

Parkinson disease (PD) is a neurodegenerative disorder characterized by both motor and nonmotor symptoms. Mild cognitive impairment can already be present in up to 40% of newly diagnosed PD patients [1] and more marked decline can ultimately be seen in up to 83% of patients [2]. Cognitive impairment is associated with a decreased quality of life, an increased caregiver burden, and an increased risk of developing dementia [3]. Unfortunately, very few treatment options are available. The only effective pharmacological treatment (rivastigmine) provides limited improvements in memory and language [4]. Recent research has pointed to small positive effects of nonpharmacological cognitive trainings on working memory, processing speed, and executive function [5-7], suggesting that these interventions could possibly attenuate cognitive deficits in PD. Many of the investigated cognitive trainings in PD are performed under supervision and include solely computerized versions of paper-pencil (traditional) cognitive trainings. These traditional cognitive trainings involve repetitive execution of cognitive tasks but lack gamification stimulants. Gamification of cognitive training can be used to promote adherence, such as reward and engagement, and could eventually improve health outcomes. Both personalization and gamification could increase the adherence to and effectiveness of cognitive training in PD. Although some previously investigated interventions adapted to the user performance, adherence variables were unfortunately insufficiently reported across these studies. Therefore, we are currently unable to conclude that gamification of cognitive training is indeed more attractive for PD patients and results in increased adherence rates. Hence, more research is needed in the area of gamified cognitive training. Before we can test the effectiveness of such gamified cognitive trainings, it would be helpful to explore whether PD patients are interested in using gamified cognitive trainings at all.

### Objectives

In this viewpoint, we have presented 3 independent histories of PD patients with different ages, with different disease stages, and from various backgrounds, who all used self-invented cognitive training that included elements of personalization and gamification. Using computer videogames, card games, or real-life routines, these patients self-trained their cognitive abilities, which are essential for activities of daily living. We will discuss the training types and present the similarities and differences between these cases. We additionally report on recruitment data from a recent gamified cognitive training trial [8]. By describing 3 different self-invented ways of cognitive gaming in patients with PD, we aimed to raise awareness for the potential of gamified cognitive training in PD patients. In addition, we hoped to inspire the readers with our case descriptions, highlighting the importance of both personalization and cocreation in the development of games for health. Finally, we have presented some considerations for future gamified cognitive training development and evaluation.

## Cases

### Case 1

**Figure 1:**
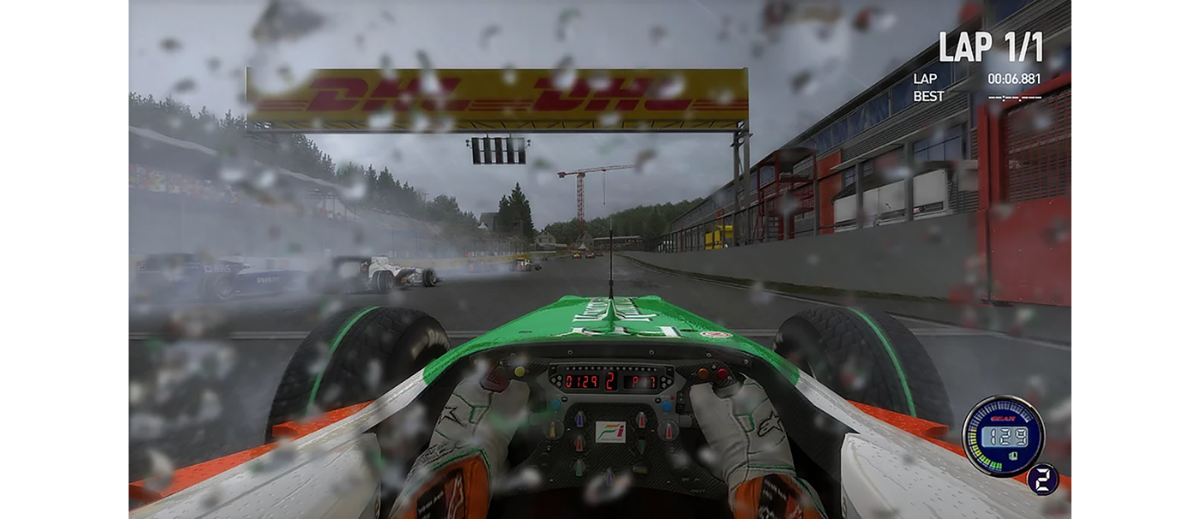
Screenshot of the racing simulator game played by Case 1.

This 64-year-old man with PD had a disease duration of 20 years and a Hoehn and Yahr Stage of III, indicating a mild-to-moderate bilateral disease and some postural instability but being physically independent (the range according to the Hoehn and Yahr stages is from 0 [no symptoms] to V [severely disabled and wheelchair bound]) [9]. In the course of his disease, he started experiencing postural instability, decreased memory performance, and depressive symptoms. His passion was virtual car racing, and he customized a computer videogame racing simulator (called iRacing, by iRacing.com Motorsport Simulations) with a trajectory on the Nürburgring Nordschleife circuit (Germany; see Figure 1 and Multimedia Appendix 1). At the time, he was treated with a levodopa equivalent daily dose of 1285 mg, including a daily dose of 3 mg Ropinirole dopamine agonist. He started racing on a daily basis in his simulator and challenged himself to improve on every race lap. He assessed his performance by remembering the influence of variances in turns on lap times. A race simulator challenges various cognitive functions (attention, decision making, and memory) as well as motor functions (reaction times and perceptuomotor skills). In the following months, he experienced improved driving skills in real life and better attentional performance while driving a real car, outside of the simulator. The patient’s spouse believed her partner had an extended attentional span after playing the game regularly. His compliance was excellent, as the pursuit of the perfect race lap on the circuit was an intrinsic motivation for creating a gamified cognitive training task. He feels that pushing the boundaries prevents a rapid cognitive decline, and he has now faithfully used his simulator for over 5 years.

### Case 2

The second case is a 67-year-old woman with PD in Hoehn and Yahr Stage II (bilateral involvement without impairment of balance) and a disease duration of 12 years. Soon after retiring as a financial consultant in the field of education, she became afraid that the decreased working load on her brain would result in memory loss. A few years into the disease, she indeed started experiencing memory loss, which motivated her to train herself in daily real-life situations. Specifically, she has developed several daily routines to train her memory. After waking up, she tries to remember all meetings for the upcoming day. She, afterward, checks her calendar to see if she was right. Also, if she is outdoors and plans a new meeting with a friend, she will note it in her calendar only by the time she comes home. Afterward, she will check to see if she remembered the correct date and time. Also, she manually enters frequently used phone numbers, even though she has saved them as contacts in her smartphone. Furthermore, when she plans on shopping for groceries, she makes a shopping list on paper that includes all the needed ingredients. In the store, however, she will not consult this list, but instead she will buy all products from memory. The shopping list is ultimately referred to as a checklist for completion. This type of real-life training requires multiple cognitive functions, including planning and memory. The patient feels that these self-invented routines keep her memory stable at an acceptable level. She is now confident that she is able to remember almost anything, and she has never heard from others that she forgot something. Importantly, compliance was again excellent, as she has been using these daily routines for over 5 years now.

### Case 3

**Figure 2:**
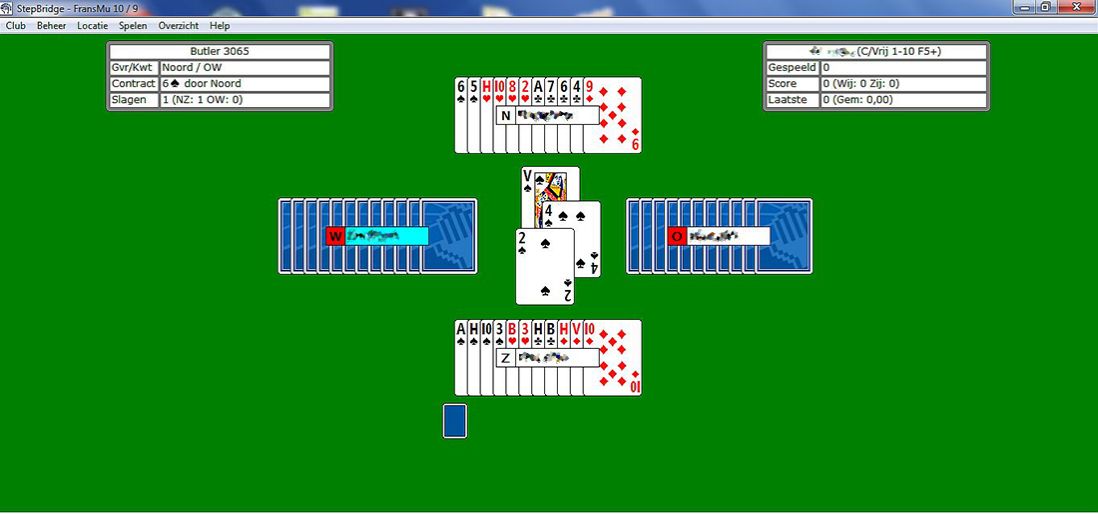
Screenshot of the StepBridge game played by Case 3.

The third case is a 68-year-old woman with PD in Hoehn and Yahr Stage III and a disease duration of 10 years. She has been living in South Africa volunteering as a community development worker for 28 years and has raised 5 children. After returning to the Netherlands, she was diagnosed with PD in 2009. In the following years, she started noticing cognitive problems, including concentration and memory deficits. She applied to a Dutch Web-based Bridge game service (called StepBridge, by StepBridge Foundation, see Figure 2), where she could play Bridge against gamers of similar difficulty levels at any time this would fit her schedule. This Bridge game requires several cognitive functions, including attention, reasoning, decision making, and memory. She reports subjective benefits in terms of both concentration and memory, which is also observed by her spouse. Compliance was again outstanding, as she has been playing StepBridge regularly for almost 10 years now.

### Generalization of These Cases

To investigate whether this interest for gamified cognitive training can be generalized to a larger PD population, a recruitment newsletter was sent out which contained information on various PD research projects. Among others, it presented a brief introduction to a randomized controlled trial on the effects of a gamified cognitive training in PD [8], including 2 clickable buttons directed to the recruitment website. The newsletter was sent on April 3, 2017 at 7 pm to 1103 PD patients in the Netherlands. As early as the next morning, 60 patients requested the patient information brochure via the recruitment website. The email was opened by over 800 patients, and the recruitment website traffic increased by over 7 times within a month. In total, 135 PD patients requested the patient information brochure via this single newsletter and 55 patients applied to the study, underlining that a larger population of PD patients may be interested in using structured and gamified ways to train cognition. The results from this study are now being analyzed and, when published, may add to the current evidence for the effectivity of gamified cognitive training.

## Discussion

### Principal Findings

The 3 patients presented here are, despite their differences in key characteristics such as age, gender, disease status, and disease duration, also similar in many ways. First, all 3 patients were aware of their decreased cognitive abilities and were proactively looking for ways to potentially counteract their imminent cognitive decline. They challenged themselves with self-invented trainings to improve their own daily life functioning. The first patient used a computer videogame race simulation to train his real-life driving skills. The second patient does not rely on lists to remember meetings or phone numbers, and thereby trains her memory performance for a variety of everyday functions such as shopping. The third patient used an online bridge game to train her concentration and memory performance. Taken together, these 3 stories carefully suggest that at least some PD patients are trying to counteract their cognitive deficits with self-invented trainings that address various cognitive skills. Whether such interventions are actually effective requires further formal testing in controlled studies.

Second, all 3 patients incorporated a form of play to address their cognitive deficits. Although the second patient did not resort to a game, the self-invented training approaches of all 3 patients entailed gamified elements (goals, challenges, and reward systems), which likely add motivation to continue the training. The first patient challenges himself to drive faster and faster laps, the second patient is rewarded each time she remembers the grocery list correctly, and the third patient has the goal to win as many tricks as possible. Indeed, in various studies, gamification has been found to increase the motivation and engagement of study subjects [10-12]. Owing to the predictability and repeatability of traditional (nongamified) trainings, eventually patients might get bored, increasing the risk of dropouts [13,14]. This could be avoided by challenging patients into performing interventions of varying complexity using attractive, interactive environments. There is debate on the support for gamified training and a large percentage of the general elderly population has never played game interventions [15], but it is unclear whether this is due to a lack of interest or if they are unfamiliar with the concept of gamified training. In exergaming studies, in the field of PD, it has already been established that patients are able to play games, improve their gameplay performance, and, more importantly, enjoy playing exergames [16,17]. One specific example was a recent study where gaming elements were used to promote adherence to a home-based exercise intervention; the results showed that PD patients, despite their well-known difficulties to engage in exercise, faithfully adhered to a regime of aerobic exercise at home, precisely as prescribed, namely 3 times a week for 30 min [18]. Also, various researchers suggested that trainings should be personalized by tailoring the intervention to the individuals’ rehabilitation needs and performance levels, thereby improving motivation and adherence [15,16,19]. All 3 cases presented above likely showed positive attitudes toward gamification and used gamification strategies, such as goal setting, reinforcement, and the capacity to overcome challenges, that have been scientifically proven to promote health behavior change and thereby influence health outcomes [20]. In addition, all 3 cases showed aspects of personalization: they chose their own way of training and made personal adjustments in gameplay or goals within their training.

Finally, all patients showed excellent adherence to the training for prolonged periods of time (several years). They were intrinsically motivated to continue, possibly because they felt that the therapy had a positive effect on their functional performance. An extrinsically motivated person requires an external reward to engage in a particular behavior, whereas intrinsic motivation arises from intrinsically rewarding factors. People may follow a training because it makes them feel better (intrinsic motivation) and feeling better may then have external benefits (extrinsic motivation) [21]. Ultimately, motivated people tend to exercise a behavior that is particularly rewarding to them, which may explain why these 3 patients continued to use their self-invented training for many years. However, Case 1 was treated with dopamine agonists, which could have resulted in increased addictive behavior in PD and thus in more adherence to the training. Nevertheless, motivation is an important influencer of adherence and it should be an important part of future interventions in this area.

### Comparison With Prior Work

All 3 patients found their own way to train their cognition, but the majority of PD patients are not likely to be able to create such self-invented trainings. However, in some patients, there seems to be a need for a structured way of training cognitive functions. Various traditional cognitive trainings have already been investigated in PD, with small-to-moderate symptomatic effects on cognition, mainly on measures of processing speed, working memory, and executive functions [5,22]. These previous studies had short follow-up periods of maximally 3 months. It would be interesting to see whether these symptomatic effects also persist in the long term and whether the progression of cognitive decline could potentially be delayed (ie, a neuroprotective or disease-modifying effect). However, none of the previous studies investigated the long-term effects on cognition in PD. To date, there is no evidence whether gamified cognitive training can suppress (let alone delay the progression of) cognitive impairment in PD. Many of the investigated cognitive trainings in PD are lacking rewarding gamification stimulants that could stimulate adherence and eventually improve health outcomes even more. In addition, many cognitive training studies had methodological challenges, such as the lack of solid sample sizes based on reliable power calculations. Importantly, showing that gamified cognitive training has disease-modifying effects that extend beyond mere symptomatic effects is very difficult and calls for specific study designs to separate temporary symptomatic improvement from a more sustained protective effect on actual progression [23].

Some efforts have been made to create gamified cognitive assessments, which may add benefits over traditional assessments in terms of reducing stress related to the formal assessment situation. These gamified assessments are usually relatively simple puzzles with, for example, added sound effects to appear as a game. More importantly, they validate well against traditional cognitive assessments [13]. Gamified cognitive assessments can additionally be used to evaluate the performance and adjust the game’s difficulty level accordingly [8]. To our knowledge, no fully gamified cognitive assessments have been investigated in PD to date.

### Theoretical Bases

Some theoretical bases have been proposed which promote health behavior when used in gamified treatments [24]. According to the self-determination theory, for example, it is assumed that everyone is driven by autonomy, competence, and relatedness [25]. Within games, autonomy can be implemented via features such as choice and structured reward systems, competence can be implemented via personalized challenges and feedback, and relatedness can be implemented via social elements [24]. For gamified treatments in PD patients, the complexity of the apathy-reward-motivation system must also be recognized [26]. Although the exact relationship is not yet clear, apathy is thought to result from dopaminergic depletion in the ventral striatum, substantia nigra, and ventral tegmental area [26]. Indeed, PD patients have a decreased reward sensitivity in an off-dopaminergic medication state [27]. Personalized trainings, with more rewarding elements and interventions that are specifically tailored to their cognitive abilities, will likely improve the self-efficacy of patients. Patients then feel more in control over the events or behaviors with regard to the training, thereby increasing motivation and enhancing resilience to failure [28]. To increase treatment adherence, a potentially ideal cognitive intervention should contain a mix of training elements targeting various cognitive domains but also contain gamified elements. In addition, it is suggested that a personalized challenge level may result in more engagement in the game [29]. Within PD, some computerized cognitive trainings have been investigated, such as RehaCom (computer-assisted cognitive rehabilitation) [30], SmartBrain (28 computerized cognitive exercises) [31], NEUROvitalis (computerized exercises training attention, memory, and executive functions) [32], and InSight (5 exercises training information processing speed) [33], but none of these trainings incorporated gamification or personalization. In other populations, positive effects have been found with health games. For example, the NeuroRacer [34], a game-like training that aims to reduce susceptibility to cognitive interference and adapts the difficulty level to the player’s performance levels (personalization), showed positive effects on attention, impulsivity, and multitasking in elderly subjects. Recently, positive results were published for the Project: Evo health game that targets cognitive conditions in children with attention deficit hyperactivity disorder (ADHD) [35]. Although Project: Evo is actually a therapy targeting specific neural circuitries involved in attentional control, the intervention feels like a videogame when it is being performed. The researchers found improvements in working memory and attention, but the treatment was also an attractive way to address ADHD, which is promising when it comes to achieving sustained treatment efficacy over time.

### Recommendations for Future Gamified Interventions

In Table 1, we briefly summarize considerations considering the design and evaluation of future gamified cognitive trainings. This table is based on recommendations from the literature on both game development and evaluation guidelines [36].

**Table 1:** Considerations for developing future gamified cognitive trainings.

Area and consideration^a^		Type
**Gameplay**		
	Adopt levels of increasing complexity (with achievable goals)	Gameplay
	Introduce cognitively demanding aspects slowly	Gameplay
	Clear user-interface design (large fonts, bright colors)	User-interface
	Include a dynamic difficulty adaptation mechanism (interactive)	Personalization
	Personalize training content to individual needs in real-time	Personalization
	Add social elements (eg, play with grandchildren)	Social functions
	Add competitive elements (against oneself, computer, or others)	Social functions
	Choose actions that are familiar to patients (daily activities)	Gameplay
	Think about fun factors (appealing story, graphics, and sounds)	Gameplay
	Set long-term goals to help sustain long-term engagement	Engagement
	Provide in-game variance (keep game engaging for longer periods)	Engagement
	Reinforce positive performance with visual/audio feedback (reward)	Feedback
	Avoid negative feedback	Feedback
	Be hesitant with negative progress reports (self-monitoring)	Feedback
**Development**		
	Integrate validated theories (eg, self-determination, motivation)	Design
	Use recent serious game development guidelines [36]	Design
	Participate with Parkinson disease patients and professionals in design/evaluation	Design
	Optionally add other neuroplasticity stimulants (eg, exercise)	Design
	(Re)evaluate the game with an evaluation protocol [36]	Evaluation
**Procedural**		
	Provide crystal-clear and guided instructions	Instructions
	Guide the patient through the first level(s)	Instructions
	Set clear goals (distinguish game targets vs training targets)	Instructions
	Adopt cross-platform availability and plug-and-play technology	Availability
	Optionally add group-based, therapist-guided booster sessions	Efficacy
**Methodological**		
	Clearly describe the training to aid in replication (publication)	Epidemiology
	Compare standardized versus personalized training	Epidemiology
	Have a solid sample size	Epidemiology
	Report standard measures of disease severity (Hoehn & Yahr Scale, Unified Parkinson’s Disease Rating Scale Part III)	Epidemiology
	Report standard measures of medication status (Levodopa equivalent daily dosage)	Epidemiology
	Report standard measures of cognitive status (Montreal Cognitive Assessment, Mini Mental State Exam)	Epidemiology
	Report objective and subjective measures of safety	Epidemiology
	Report measures of feasibility and adherence	Epidemiology

^a^Noncomprehensive considerations for gamified cognitive training design (in the field of Parkinson disease); not presented in order of priority and obtained from the wider literature [7,13,15,16,19,20,30,36-39].

### Conclusions

Taken together, the 3 patients presented here as well as the recruitment results from a gamified and personalized cognitive training trial [8] may justify the development of more structured ways of training cognitive functions in PD, while incorporating elements to increase adherence such as personalization and gamification. Positive results have already been found with gamified trainings in other populations, and the cases described here suggest that PD is also an attractive area to develop and test gamified cognitive trainings. Our 3 patients also demonstrate enormous creativity and laudable resilience despite having PD. However, the majority of PD patients are not likely to be able to create such self-invented trainings. Researchers, health professionals, patients, and the industry should therefore collaborate to develop motivating and targeted cognitive trainings for persons with PD, for which our considerations offered here can be used. The first steps in this direction have already been taken, and several trials are now ongoing [8,18,40,41].

## Appendix

Multimedia Appendix 1 Short clip of Case 1 training in the racing simulator game.

## Abbreviations

ADHD: attention deficit hyperactivity disorder
PD: Parkinson disease
